# Development and technical application of SSR-based individual identification system for *Chamaecyparis taiwanensis *against illegal logging convictions

**DOI:** 10.1038/s41598-020-79061-z

**Published:** 2020-12-16

**Authors:** Chiun-Jr Huang, Fang-Hua Chu, Yi-Shiang Huang, Yu- Mei Hung, Yu-Hsin Tseng, Chang-En Pu, Chi-Hsiang Chao, Yu-Shyang Chou, Shau-Chian Liu, Ya Ting You, Shuo-Yu Hsu, Hsiang-Chih Hsieh, Cheng Te Hsu, Meng-Yi Chen, Ting-An Lin, Hsin-Yi Shyu, Yu-Ching Tu, Chi-Tsong Chen

**Affiliations:** 1 School of Forestry and Resource Conservation, National Taiwan University, Taipei, 10617 Taiwan; 2 Biodiversity Research Center, Academia Sinica, Taipei, 11529 Taiwan; 3 Department of Forensic Science, Investigation Bureau, Ministry of Justice, New Taipei City, 23149 Taiwan; 4 Institute of Biological Chemistry, Academia Sinica, Taipei, 11529 Taiwan; 5 Department of Research Committee, Investigation Bureau, Ministry of Justice, New Taipei City, 23149 Taiwan; 6 Department of Applied Science, National Taitung University, Taitung, 95092 Taiwan; 7 Hualien Forest District Office, Forestry Bureau, Council of Agriculture, Hualien, 97051 Taiwan

**Keywords:** Biotechnology, Plant sciences

## Abstract

*Chamaecyparis taiwanensis* is an endemic plant suffering illegal logging in Taiwan for its high economic value. Lack of direct evidence to correlate stump and timber remains a hurdle for law enforcement. In this report, 23 polymorphic Genomic Simple Sequence Repeat (gSSR) and 12 Expressed Sequence Tag (EST)-SSR markers were developed and their transferability was assessed. The individual identification system built from selected non-linkage 30 SSR markers has a combined probability of identity as 5.596 × 10^–12^ equivalents to identifying an individual in a population of up to 18 million *C. taiwanensis* with 99.99% confidence level. We also applied the system in an actual criminal case by selecting 19 of these markers to correlate illegally felled timbers and victim trees. Our data demonstrate that molecular signals from three timbers hit with three victim trees with confidence level more than 99.99%. This is the first example of successfully applying SSR in *C. taiwanensis* as a court evidence for law enforcement. The identification system adapted advanced molecular technology and exhibits its great potential for natural resource management on *C. taiwanensis.*

## Introduction

*Chamaecyparis taiwanensis* Masam. & Suzuki [= *Chamaecyparis obtusa* (Sieb. & Zucc.) Endl. var. *formosana* (Hayata) Hayata] (Cupressaceae) is a gymnosperm endemic in Taiwan. *C. taiwanensis* is endemic to Taiwan and is the dominant species in the conifer and broadleaf tree mixed forest, located in middle altitude region (from 1700 m to 2600 m) of Taiwan island^1^. The lowest latitude boundary of cypress’ natural distribution falls into Taiwan, suggesting a great significance in biogeography^2^. As an indispensable resource for making elegant buildings, furniture and handicrafts, these species play a vital role in serving wood source and timber industry. *C. taiwanensis* is well-known for their wood quality and expensiveness (4400 USD/m^3^)(woodprice.forest.gov.tw), which often lead to endless illegal felling crimes. Therefore, developing individual identification system to *C.* *taiwanensis* is of more importance^3^.

Illegal felling remains a persistent problem in the timber producing countries all over the world. For decades, illegal logging endangered precious and valuable tree species such as cypress^4^, ash^5^, mahogany tree^6^, and Brazilian rosewood^7^ all over the world. In some cases, the law enforcement authorities, such as forestry police, arrest the suspects in time. However, lack of direct scientific evidence that correlate timbers to the stumps leads the conviction processes rather difficult and ineffective. Thus, the need of individual identification is critical to the forestry industry.

The problem of illegal logging has been paid attention since 1995. More and more national and international regulations mandate tracking systems that ensure traceability on wood market^8–10^. Wood anatomy and dendrochronology are common visual identification method. The former is based on the anatomical characteristics to identify the wood, and can usually be identified to the genus^11^; the latter is often used to illustrate past climates, but may also provide the age and origin of the trees^12^. Compounds synthesized by trees and other plants are often called phytochemicals and are often used to identify species or distinguish genera. Intraspecific variation can also be detected in some species through some chemical analysis such as mass spectrometry^12,13^, near infrared spectroscopy^14^, detector dogs^15^, stable isotopes^16^, and radiocarbon^17^. Genetic analysis can provide species-level identification, which is usually achieved by DNA sequence polymorphism^18^. Simple sequence repeats (SSRs) and Single nucleotide polymorphisms (SNPs) can be used to identify individuals and can be used in population genetics or systematic geography to determine the geographical region of origin within a species^19^. DNA fingerprinting is built into each organism itself and cannot be forged^20^. When enough markers are developed, in principle every individual has its own unique DNA fingerprint. DNA fingerprinting has the potential to track wood products independently within complex global supply network^21^. Theoretically, DNA fingerprinting is the only forensic wood identification technology that could be used to connect seized timber to illegally felled stumps^8^.

SSR is the most common marker used in individual identification for its short length, high polymorphism, easy polymerase chain reaction (PCR) amplification, high reproducibility, and high sensitivity^20,22,23^. SSRs are divided into two broad categories by different sources: Genomic (g)-SSR and expressed sequence tag (EST)-SSR^24^. gSSR markers are derived from amplified genomic libraries. EST-SSRs are markers mined from EST sequence collections. gSSR markers have been reported to be more polymorphic when compared with EST-SSR in gymnosperms^4^ and crops^25,26^ because of a more diversified nucleotide sequence. Since the development of high-throughput sequencing technology, the marker development technique has been continuously advanced. Wang et al., 2018 published the first report on gSSR developed by De novo genome sequencing^27^. In contrast, EST-SSR, derived from the expressed sequence, is fast-acting, cost-effective and labor-saving alternative for non-model organisms^24^. Because of the conservative nature in gene coding regions^24^, newly developed EST-SSRs usually can be transferred in closely related species for marker development. The first EST-SSR based on Illumina-based de novo transcriptome was also published by Zhou et al. in 2018^28^. A study to develop both markers would avail of their merits and functions simultaneously.

For *C. taiwanensis*, evaluation of genetic variation or population structure is necessary for its preservation^2,29^ because this species is used extensively. After mid-twentieth century, the number of *C. taiwanensis* plunged, which also led to a significant decrease in both genetic variation and population structure. As an important tool for genetic and subsequent breeding, SSR markers are helpful for breeding polymorphic maternal plants and increasing the diversity of progeny. The objective of this study is to establish a scientifically valid SSR mediated individual identification system for *C. taiwanensis* in order to provide court evidence to link the seized wood and the victim tree, and to provide traceability proof for wood supply network. In the beginning of the research, we used Next Generation Sequence (NGS) technology to establish the DNA and RNA libraries of *C. taiwanensis* to accelerate the development of gSSR and EST-SSR markers. A total of 96 samples from four populations were used to evaluate the polymorphism, discriminative power, and random match rate of the selected SSRs. The linkage disequilibrium between markers was calculated to estimate the availability and credibility of the individual identification system. In this study, we successfully linked 3 stolen timbers back to 3 victim trees (case number MJIB-DNA-1080413 combine 1080328), marked the first successful application of *C. taiwanesis* individual identification system. Finally, our work would deter illegal felling toward these precious species by manifesting law enforcement effectively.

## Result and discussion

### Developing *C. taiwanensis* individual identification system

#### Choice of template and library preparation

**Figure 1:**
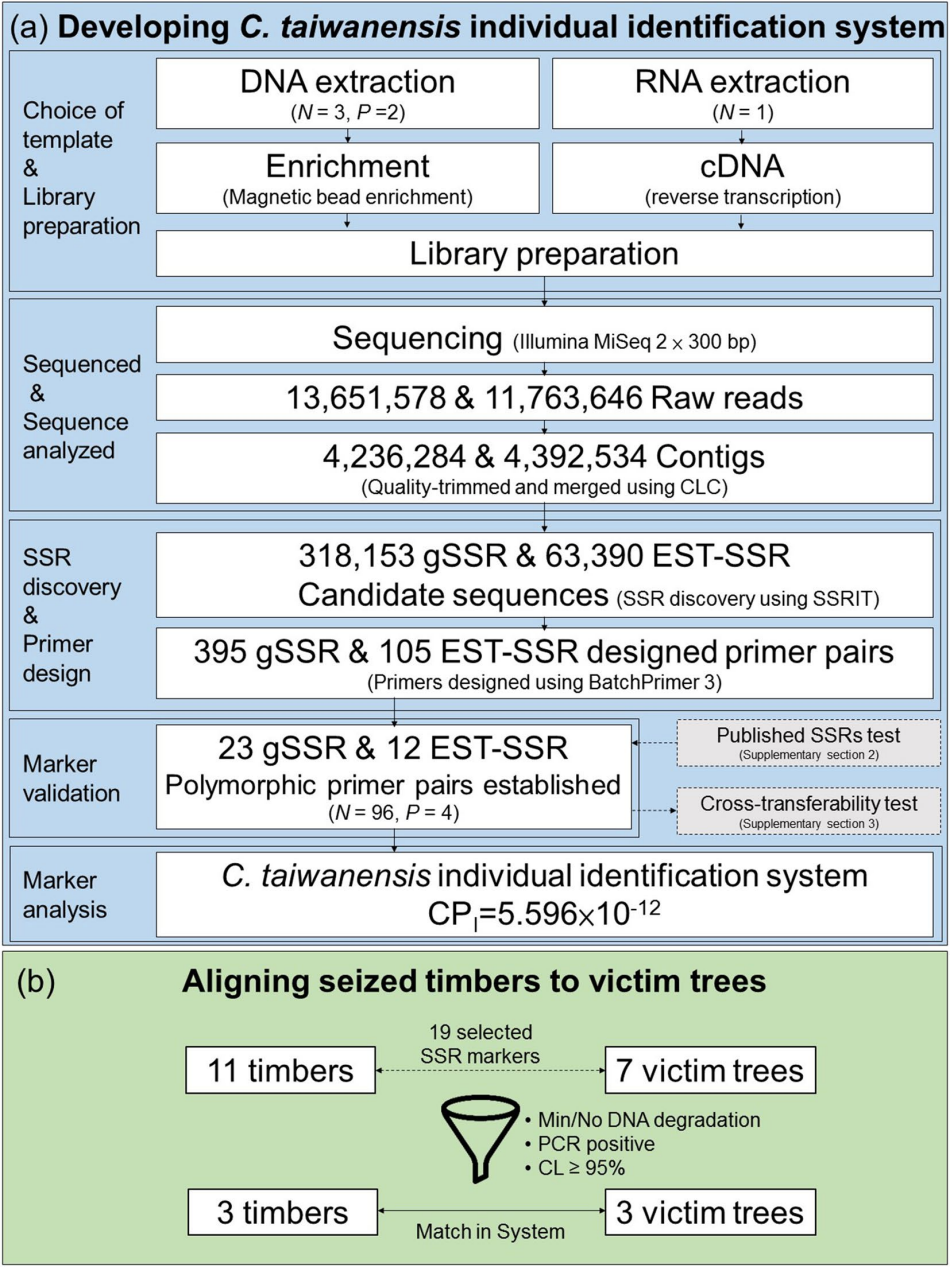
Flowchart describing the procedure of developing SSR markers and aligning illegally-felled timbers to victim trees of *C. taiwanensis*. (**a**) 35 SSR markers specific to individual identification were selected from the DNA and RNA libraries of *C. taiwanensis*. The cumulative random matching rate of the system reaches CP_I_ = 5.596 × 10^–12^, which can be used to identify 18 million individuals with a credibility of 99.99% (**b**) 11 seized timbers were compared with 7 victim trees, and 3 timbers were matched with 3 victim trees successfully. The values of credibility in all matched cases were over 95%. (*N *number of individuals, *P *number of populations).

The gSSR are characterized by high polymorphism and is suitable for developing individual identification markers. The EST-SSR are highly conservative which could be used for developing markers to categorize species and populations^20,22,23^. In this study, both DNA and RNA libraries were constructed simultaneously as gSSR and EST-SSR markers, respectively (Fig. 1, Supplementary Sect. 1). From the three DNA libraries and from a RNA library prepared for the study, the sequences were compared between individual plants as well as between groups (Supplementary Sect. 1). With these two nucleic acid markers, we envisioned to differentiate samples within or among species.

#### Nucleic acid sequencing and analysis

Next-generation sequencing technology enables the possible procurement of large number of sequences in a short time. In this study, we used the Illumina MiSeq platform (2 × 300 bp) to sequence the DNA and RNA libraries (Fig. 1). A total of 13,651,578 and 11,763,646 raw reads were produced from DNA and RNA libraries, respectively. The raw reads were deposited in the NCBI Sequence Read Archive (PRJNA506084). The sequences were then subjected to quality-trimming and merging and afterwards 4,236,284 contigs of the DNA pool and 4,392,534 RNA contigs were assembled. The base lengths of contigs ranged from 120–579 and 120–529, at an average of 420 for DNA and RNA, respectively. According to the work published by timber researchers^23,30^, the nucleic acid markers with fragment lengths of around 250 bases best meet our research goals. The lengths of contigs derived from the four libraries we have prepared were found to be suitable for screening markers within 250 bp length. A target band size below 250 bp implies a higher PCR success rate as the DNA of wood samples from seized timber and victim trees were mostly severely degraded.

#### SSR discovery and primer design

A sum of 318,153 gSSR and 63,390 EST-SSR candidate sequences were screened by Simple Sequence Repeat Identification Tool (SSRIT)^31^ (Fig. 1). The proportions of SSR in the genomic DNA and RNA libraries were 7.51% and 1.44%, respectively. Study by Squirrell et al.^32^ suggests that the overall success rate of SSR marker development is about 10%. With PCR, polymorphic high-quality markers could be successfully amplified resulting to a good peak pattern quality with little stuttering and absence of non-amplifying (null) alleles and other factors. Therefore, about 90% of the designed markers could be screened out. We designed a total of 395 gSSR and 105 EST-SSR primer pairs for testing in *C. taiwanensis*.

#### Marker validation

From the PCR results, 23 gSSR and 12 EST-SSR markers with polymorphism were selected (Fig. 1, Tables 1, 2), and the success rate for gSSR and EST-SSR marker was found to be 5.82% and 11.42%, respectively. Our data showed that it is easier to select SSR markers from the RNA library than from the DNA library, which is akin to previous studies^4,32,33^. Other reports^24,34,35^ suggest that SSR occurs more frequently in EST sequence than in the genome. In addition, the fact that the information content in EST is markedly lower than that in the genome promotes the calculation and analysis of EST in silico^24,33^.

**Table 1:** Characteristics of 23 gSSR loci developed in *Chamaecyparis taiwanensis*.

Locus	Primer sequences (5′–3′)	Repeat motif	Fluorescent label	Allele size (bp)	*Ta* (℃)	GenBank accession no.
CoTW76	F: TCTCATTCAAGTGGTATGTT R: TCATCTTCACGAACCAAGA	(TATC)_16_	JOE	161	TD58-55	MK213959
CoTW77	F: TGACGTGTCAATCTTTTGG R: AAGAAAAGGTTGCAATGGT	(TATC)_7_	FAM	170	TD58-55	MK213960
CoTW99	F: GGGAGCTGTAGGGAGATGAA R: ACATTGCAAATAGGGGTATG	(TATG)_6_	JOE	165	TD58-55	MK213961
CoTW314	F: TGTTGACATCAATAACAATCACTA R: GGGCATGATAATGTAAAGATG	(ACA)_7_	FAM	105	52.5	MK213962
CoTW330	F: CCTAAGGTAGCAGGAATGAG R: TCTCCACTCTAGACCTAGTTTAT	(GAT)_10_	FAM	128	52.5	MK213963
CoTW337	F: CCACCTTGTACTCTAGATCCTC R: GATTCCACTAAGCCTTTCCT	(CAT)_13_	FAM	107	57.5	MK213964
CoTW349	F: GCTTGGTCATTTGTTTCATT R: TCAACTGCATTACCCAAACT	(CTT)_10_	FAM	91	57.5	MK213965
CoTW495	F: TTTCGAAATCAACATTATGCAA R: TCATTTCTCTCAAAGGGTTGAA	(ATAC)_4_	FAM	160	45	MK213966
CoTW531	F: CCCAACCACATTTACAAAATA R: TTTGTGGCTTTTTGAATAGAG	(AAAT)_5_	FAM	166	55	MK213967
CoTW539	F: AACCTCTTCCACCAATGTAAT R: TTACGTTTTCTTGGTCTAGCA	(AAAT)_5_	JOE	152	60	MK213968
CoTW545	F: GGAGGAAAGTGTTGAATCTCT R: CATAGTTGGGTTTTCACCTTC	(AAAT)_5_	JOE	151	60	MK213969
CoTW554	F: ATTTTAAAAGCTAACCCCAAC R: TCAAGCTAGAGGTTGTTCAAG	(AAAT)_5_	JOE	139	55	MK213970
CoTW556	F: GGAGAGTACCTTGGTTTATCCT R: TTTGAGATTGGCAGTATTTAGA	(AAAT)_4_	JOE	153	55	MK213971
CoTW559	F: CACCTGAACTAGAGGACAAAA R: TGTTCACCTAGCTCATTCCTA	(AAAT)_4_	JOE	150	57.5	MK213972
CoTW561	F: ATAAAGGGATTCAATGGCATA R: GGCTCCTTTATTGTTGGTATAA	(TAAT)_4_	JOE	169	55	MK213973
CoTW582	F: CTCATGGACCTGATTTCATAG R: GGAAAAACACATATGCATCAA	(AC)_13_(ATGT)_5_	JOE	200	57.5	MK213974
CoTW585	F: TCCTCATAACTAATGACAACGA R: AGGGAACGTATCCTTTAGAGA	(CA)_9_(ATGT)_4_	NED	160	57.5	MK213975
CoTW588	F: CAGGTCCTTGTAAAACCTCTC R: TGCGTGCATACATACATACAT	(AT)_4_(TATG)_7_(ATGT)_9_	NED	167	57.5	MK213976
CoTW595	F: ATGTATGCATGTATGTATGTGT R: CCCTCTTGCCTCTTTTATCTA	(TGTA)_13_	NED	125	60	MK213977
CoTW597	F: CGTATGTATGTATGTATGTATGG R: TGATTGACCCTCATAGAGTTG	(GTAT)_5_(ATGT)_9_(TATG)_4_	NED	169	55	MK213978
CoTW598	F: TCTTGCTCTTCAAATTAGCTG R: GGCAAGTGAGCATTACAAAT	(ATGT)_6_	NED	264	55	MK213979
CoTW599	F: TGCAACAATAAGAAATGGACT R: TACATGGTTGGATTGTCCTTA	(GT)_7_(ATGT)_5_(ACAT)_12_(AT)_4_	NED	166	60	MK213980
CoTW600	F: TGTGTATGTTTACGTGTACGTTT R: ACAAAATCTTCTTATCAACACG	(TGTATG)_9_(ATGT)_8_	NED	184	55	MK213981

*Ta *annealing temperature, *TD *touchdown PCR.

**Table 2:** Characteristics of 12 EST-SSR loci developed in *Chamaecyparis taiwanensis*.

Locus	Primer sequences (5′–3′)	Repeat motif	Fluorescent label	Allele size (bp)	*Ta* (℃)	GenBank accession no	Putative function [organism]
CoTW383	F: GATAGCCAGCCATATTTTTG R: GATCAAAATGGCCCTACTAT	(GAAGC)_10_	FAM	113	57.5	MK213982	Hypothetical protein (chloroplast) [*Hydrodictyon reticulatum*]
CoTW407	F: AGCACAACAGCTGGTTTATAG R: TCTATTAGTTGTGTTTGATCACCT	(GATG)_5_	FAM	75	55	MK213983	No hit
CoTW409	F: TTGGATGTAGGGAACAAGAG R: AATCAGCCACCATCACTATC	(ATAG)_10_	FAM	81	60	MK213984	No hit
CoTW420	F: CATCTAAGTGTGCTGACCACAAG R: CAGCGAGACACGATTCAGG	(ATAG)_8_	FAM	101	55	MK213985	No hit
CoTW424	F: CACTGGTGATCTTTGAACTAGGG R: CAACACACATCACGGGTACA	(ATCT)_6_(AT)_5_	FAM	100	55	MK213986	No hit
CoTW502	F: GTTTGACTGGTTTTAGGGAAG R: TGGGGTCATTGATTTAGTAGA	(AAAT)_5_	FAM	154	55	MK213987	No hit
CoTW504	F: CCCGCACAGACAGTATAAAAT R: TCTAATGTTGTGTGGTGGTTT	(AAAT)_9_	FAM	159	55	MK213988	No hit
CoTW511	F: AAGAACCAAGAGATGTCATTTT R: CTAGCTACAGGGAATTTTCGT	(AAAT)_5_	FAM	138	50	MK213989	No hit
CoTW513	F: TGGAGAATAATCAACTTCATC R: AGTGGTATTAAGGGATATCGAC	(AT)_10_	FAM	281	55	MK213990	No hit
CoTW514	F: GCAGCAGAATTTGATGATAATA R: TTCCTTGTCCAAGCATATTTA	(AAAT)_5_	FAM	291	55	MK213991	No hit
CoTW528	F: CCTTCGAATACAATCATCTCA R: GCCCAAAAACATTAAAAACTC	(AAAT)_5_	FAM	147	55	MK213992	No hit
CoTW581	F: TGAAGGATGGTAGTAATGCTC R: ACATTCTCACTTGCATGAGTT	(TA)_18_(ATGT)_8_	JOE	196	57.5	MK213993	Hypothetical protein BS50DRAFT_412996 [*Corynespora cassiicola* Philippines]

*Ta* = annealing temperature.

The samples used in marker validation came from 4 ethnic groups (TP, SY, DS, FR), with 20 to 30 individuals in each group (*N* = 25, 29, 21, 21), qualified the basic requirement of at least 15 individuals per group and 3 groups per study (Fig. 1, Supplementary Sect. 1). Among the 96 individuals sampled in this study, the number of alleles per gSSR is between 2 and 14 with 6.5 in average, whereas the number of alleles per EST-SSR is between 2 and 16, 7 in average (Tables 3, 4). The levels of *Ho* are from 0.000 to 0.802 and 0.021 to 0.604, with average of 0.399 and 0.379, respectively. The levels of *He* of gSSR and EST-SSR are ranged from 0.041 to 0.833 and 0.205 to 0.872, with average of 0.488 and 0.528, respectively. Significant (*P* < 0.001) deviations of Hardy–Weinberg equilibrium (HWE) in terms of heterozygosity deficiency were detected in 9 gSSR loci: CoTW76, CoTW77, CoTW539, CoTW545, CoTW554, CoTW556, CoTW561, CoTW585 and CoTW595 (9/23 = 39.13%) and also in 6 EST-SSR loci: CoTW383, CoTW502, CoTW511, CoTW513, CoTW514 and CoTW528(6/12 = 50%). The levels of PIC of gSSR and EST-SSR are ranged from 0.058 to 0.821 and 0.187 to 0.858, with average 0.459 and 0.482. The levels of PD from 0.041 to 0.749 and 0.205 to 0.885, with average 0.494 and 0.555. The levels of PE of gSSR and EST-SSR are ranged from 0.000 to 0.479 and 0.000 to 0.312, with average 0.169 and 0.180. The levels of P_I_ of gSSR and EST-SSR from 0.029 to 0.939 and 0.114 to 0.794, with average 0.505 and 0.443. Two EST-SSR markers, CoTW383 and CoTW581, have putative functions found by BLAST hit (Table 2). Heterozygosity, being one of the first parameters that appear often in a data set, reveals lot of information including population structure and other historical clue. High heterozygosity means a lot of genetic variation, whereas low heterozygosity means almost no genetic variation. The heterozygosity data echo the results of PIC, PD and PI, suggesting that the SSR marker developed in this study has moderate genetic variation. In addition, most of these markers show Ho < He (except CoTW495, CoTW556, CoTW559, CoTW598, CoTW424), which suggests that the population of *C. taiwanensis* is an inbred. A total of 15 sets of SSR marker used in this study deviated from HWE, which suggest the population may be not under the ideal status of HWE. The reason for this deviation could be artificial selection, non-panmixia or genetic drift^36^. Generally, EST-SSR markers are less polymorphic than gSSR in plants because of high conservation in transcribed regions^24^. Moreover, other factors^33,37^ such as SSR motif type, sample size, population and species may also differentiate gSSR and EST-SSR markers. However in this study, in terms of polymorphism and cross-species transferability, there was no significant difference between gSSR and EST-SSR groups (Supplementary Sects. 2, 3), but the difference rather occurred among individual markers. This fact might be explained by polymorphism and detection limit as markers with higher PD are often selected for individual identification. Also in our study, the differences in polymorphism and cross-species transferability between gSSR and EST-SSR are not significant, but those among markers are significant. It might be because of the giant genome size in taxa *Chamaecyparis* (20.03–27.40 pg/2C)^38,39^ which leads a deviation from random sampling in marker selection. When using the system to perform individual identification assay, a marker with higher PD should be considered as priority.

**Table 3:** Genetic characterization of 23 polymorphic gSSR loci of *Chamaecyparis taiwanensis*.

Locus	A	*Ho*	*He*	PIC	PD	PE	P_I_	*N*
CoTW76	9	0.411	0.670*	0.644	0.670	0.151	0.329	95
CoTW77	4	0.022	0.477*	0.476	0.476	0.000	0.523	93
CoTW99	6	0.333	0.378	0.354	0.378	0.102	0.621	96
CoTW314	5	0.396	0.492	0.420	0.492	0.141	0.507	96
CoTW330	9	0.604	0.642	0.576	0.642	0.312	0.357	96
CoTW337	10	0.625	0.662	0.641	0.662	0.333	0.337	96
CoTW349	9	0.615	0.626	0.591	0.626	0.322	0.373	96
CoTW495	5	0.427	0.414	0.371	0.414	0.163	0.585	96
CoTW531	4	0.083	0.119	0.115	0.119	0.006	0.880	96
CoTW539	2	0.000	0.170*	0.155	0.169	0.000	0.830	96
CoTW545	2	0.000	0.041*	0.059	0.041	0.000	0.958	95
CoTW554	3	0.042	0.452*	0.381	0.452	0.001	0.547	96
CoTW556	4	0.747	0.605*	0.548	0.605	0.479	0.394	95
CoTW559	2	0.479	0.375	0.304	0.375	0.202	0.625	96
CoTW561	2	0.000	0.061*	0.058	0.060	0.000	0.939	96
CoTW582	14	0.635	0.737	0.714	0.736	0.344	0.263	96
CoTW585	7	0.531	0.546*	0.514	0.546	0.240	0.453	96
CoTW588	10	0.615	0.719	0.684	0.719	0.322	0.280	96
CoTW595	9	0.802	0.833*	0.821	0.970	0.002	0.029	96
CoTW597	9	0.583	0.681	0.639	0.680	0.292	0.319	96
CoTW598	4	0.277	0.257	0.260	0.256	0.072	0.743	94
CoTW599	12	0.542	0.749	0.715	0.749	0.245	0.250	96
CoTW600	9	0.426	0.527	0.519	0.526	0.162	0.473	94
Average	6.521	0.399	0.488	0.459	0.494	0.169	0.505	

*A* number of alleles, *Ho* observed heterozygosity, *He* expected heterozygosity, *PIC* polymorphism information content or power of information content, *PD* power of discrimination, *PE* power of exclusion or probability of exclusion, *P*_*I*_ probability of identity, PD is equal to 1 − P_I_, *N* number of individuals.

*Highly significant from Hardy–Weinberg equilibrium (*P* < 0.001).

**Table 4:** Genetic characterization of 12 polymorphic EST-SSR loci of *Chamaecyparis taiwanensis*.

Locus	A	*Ho*	*He*	PIC	PD	PE	P_I_	*N*
CoTW383	12	0.104	0.872*	0.858	0.872	0.010	0.127	96
CoTW407	6	0.448	0.475	0.389	0.474	0.178	0.525	96
CoTW409	16	0.458	0.629	0.611	0.629	0.186	0.370	96
CoTW420	4	0.323	0.388	0.365	0.387	0.096	0.612	96
CoTW424	3	0.552	0.505	0.382	0.504	0.263	0.495	96
CoTW502	3	0.219	0.327*	0.298	0.326	0.046	0.673	96
CoTW504	4	0.604	0.622	0.577	0.622	0.312	0.377	96
CoTW511	3	0.021	0.205*	0.187	0.205	0.000	0.794	96
CoTW513	5	0.563	0.721*	0.672	0.720	0.263	0.279	96
CoTW514	4	0.292	0.481*	0.443	0.481	0.079	0.518	96
CoTW528	3	0.448	0.499*	0.419	0.498	0.178	0.501	96
CoTW581	12	0.521	0.615	0.583	0.614	0.231	0.385	96
Average	7.000	0.379	0.528	0.482	0.555	0.180	0.443	

*A* number of alleles, *Ho* observed heterozygosity, *He* expected heterozygosity, *PIC* polymorphism information content or power of information content, *PD* power of discrimination, *PE* power of exclusion or probability of exclusion, *P*_*I*_ probability of identity, PD is equal to 1 − P_I_, *N* number of individuals.

*Highly significant from Hardy–Weinberg equilibrium (*P* < 0.001).

### Probability of identity and power of discrimination analysis

Continued multiplication can be used to calculate the cumulative random probability of identity (CP_I_) and the combined power of discrimination (CPD) for non-linked markers, where CP_I_ is the probability of two individuals most likely the same genotype, CPD is the probability of individuals being identified, and CP_I_ + CPD = 1. The credibility of the system is calculated based on "Random match probability in population size and confidence levels" published by Budowle et al.^43^: Confidence levels = (1 − CP_I_)^*N*^ where *N* = Population size.

While applying the system in criminal cases, for the sake of objective and impartiality, practically the court will use the credibility of 95%, 99%, or 99.99% as aacceptance criteria^40^ (Wall 2002, ISO ISO/IEC 17025). In this study, only one marker in the same linkage group is used for CP_I_ analysis, and up to 30 markers can be continuously accumulated (Table 5). Also, the individual identification system’s CP_I_ is as small as 5.596 × 10^–12^, and the CPD is as high as 0.99999999994404 (extremely close to 1). Applying the court's strictest credibility standard of 99.99%, when the number of markers reached up to 30, the system can identify 18 million individuals, which actually exceed the whole *C. taiwanensis* population of 7.39 ± 0.73 million in Taiwan^41^. While applying to the lowest acceptable credibility standard of 95%, the system could identify at least 2300 plants with a minimum number of 6 markers (Table 5).

**Table 5:**
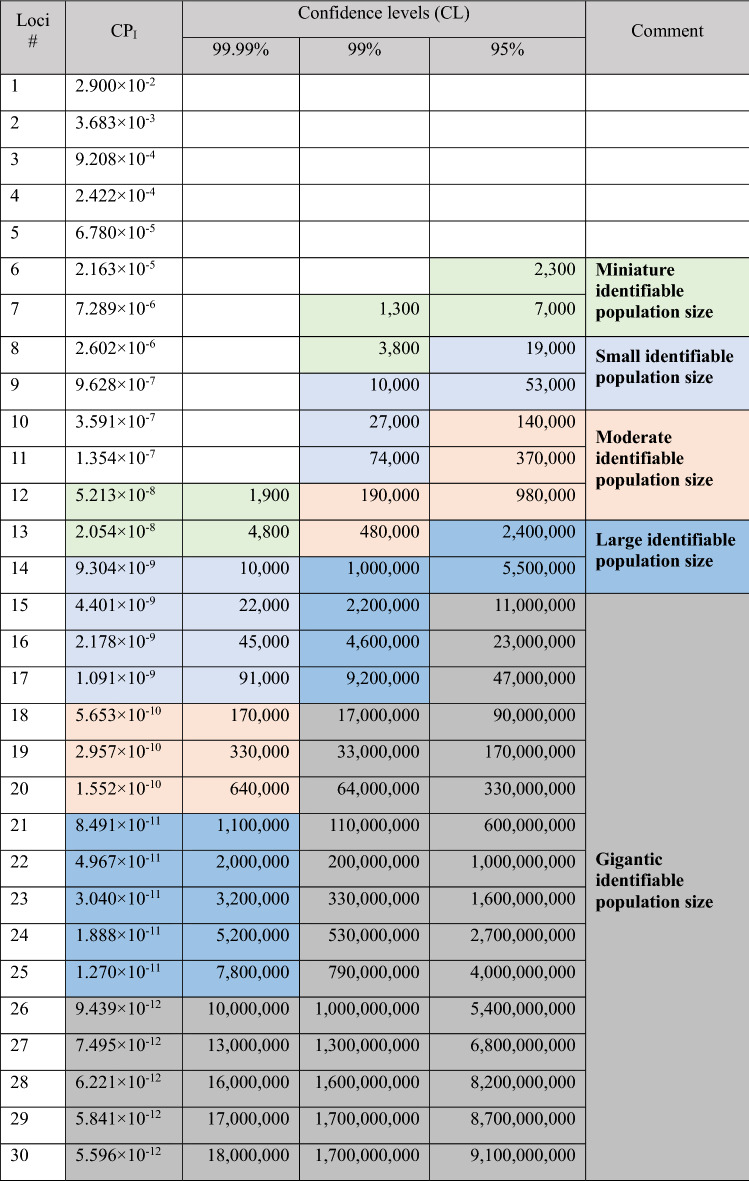
The discrimination power in SSR marker combination.

*CP*_*I*_ cumulative random probability of identity, CL = (1 − CP_I_)^*N*^, *N* number of individuals.

### Aligning seized timbers to victim trees

**Figure 2:**
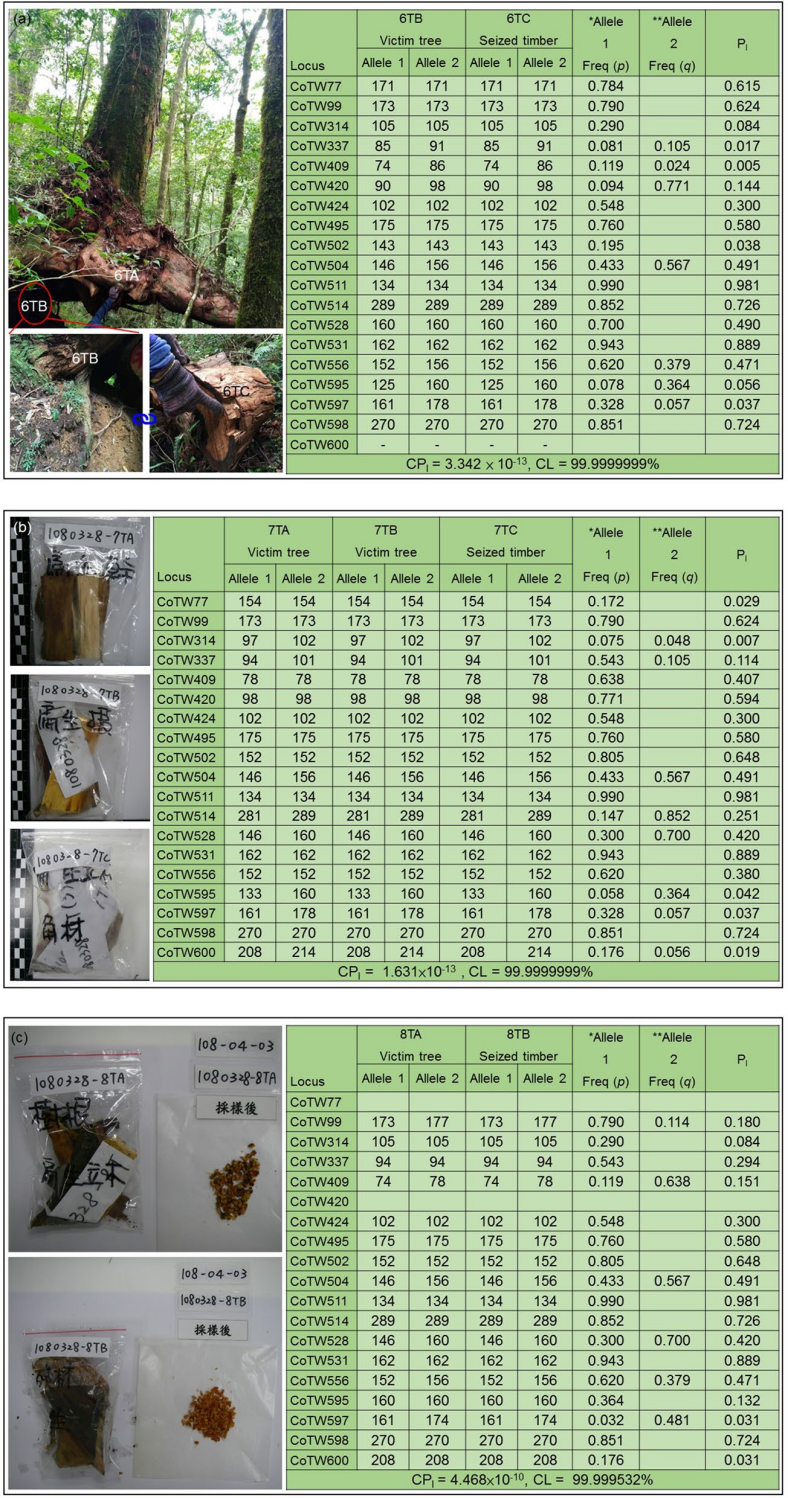
The photos and matrices of the three groups in which seized timbers and victim trees are successfully linked via 19 non-linked SSR markers matches. (**a**) Group 6TB/6TC, in wild. (**b**) Group 7TA/7TB/7TC, sampled. (**c**) Group 8TA/8TB, chopped. *A1 Freq Allele 1 Frequency. **A2 Freq = Allele 2 Frequency. Allele frequencies are from 96 individuals of *C. taiwanensis*. P_I_ = *p*^2^ or 2*pq*. *CP*_*I*_ combined probability of identity, *CL *confidence levels.

In this case (MJIB-DNA-1080413 combine 1,080,328), we successfully matched 3 seized timbers back to 3 victim trees by using 19 pairs of non-linkage SSR markers (Fig. 2, Table 6, Supplementary Sect. 4). The credibility values of the 3 cases are all above 99.99%. In our experiments, DNA samples were extracted twice or more from each sample in order to optimize the DNA concentration. Since 2007, forestry researchers have noticed that molecular markers can be used to provide direct evidence linking stolen timber and victim trees^42^. Although many techniques have developed for extracting DNA from fresh and dried leaves (including published literature^43,44^ and commercial reagents), yet few studies have reported on extracting DNA from dried wood, which is still considered the most challenging part in this field of research^45^. In forensic science field of study, it has been established that the validated DNA concentration range is between 0.625 and 10 ng/μL^46^. False negative result cannot be ruled out from over-concentrated sample and vice versa. Therefore, it is necessary to extract DNA two or more times for dry timber, as abovementioned, because its DNA extraction is challenging. Several studies suggested that the error rate increases along with PCR cycles^47,48^. Base misincorporation incurred by PCR occurs randomly throughout the sequence without hot spots^48^. The probability of base misincorporation is 1.85 × 10^–5^ per base per cycle^48^. After comparing the results of positive and negative endpoint, we discovered 36 cycles is the upper limit which leads to positive PCR product without false-positive result. From comparing the results of positive and negative endpoint, we discovered 36 cycles is the upper limit which leads to positive PCR product without false-positive result. Therefore, the cycles were controlled below 35 cycles in our study, but not increasing cycles without limit. In addition, the SSR types of each marker were analyzed at least twice with ABI 3130XL. Signals below 150 RFU peak height threshold were considered not detectable. We developed a protocol of two sessions of instrumental operation and setup threshold value from pilot test result for illegal felling investigation cases. By comparing the profiles from positive and negative controls with test samples, we can obtain objective data with least erroneous possibility to conclude our investigation for court evidence.

**Table 6:** individual P_I_ and CP_I_ of four matched groups.

Locus	Matched samples		
	6TB 6TC	7TA 7TB 7TC	8TA 8TB
	^a^P_I_		
CoTW77	0.615	0.029	
CoTW99	0.624	0.624	0.180
CoTW314	0.084	0.007	0.084
CoTW337	0.017	0.114	0.294
CoTW409	0.005	0.407	0.151
CoTW420	0.144	0.594	
CoTW424	0.300	0.300	0.300
CoTW495	0.580	0.580	0.580
CoTW502	0.038	0.648	0.648
CoTW504	0.491	0.491	0.491
CoTW511	0.981	0.981	0.981
CoTW514	0.726	0.251	0.726
CoTW528	0.490	0.420	0.420
CoTW531	0.889	0.889	0.889
CoTW556	0.471	0.380	0.471
CoTW595	0.056	0.042	0.132
CoTW597	0.037	0.037	0.031
CoTW598	0.724	0.724	0.724
CoTW600		0.019	0.031
CP_I_	3.342 × 10^–13^	1.631 × 10^–13^	4.468 × 10^–10^
CL	99.9999999%	99.9999999%	99.999532%

*P*_*I*_ probability of identity, *CP*_*I*_ combined probability of identity, *CL* confidence levels.

^a^Database: 96 *C. taiwanensis* individuals. Markers failed in PCR amplification or their peak heights smaller than RFU150 in typing were not subjected into P_I_ calculation and are shown in blank. 6TB, 7TA, 7TB and 8TA are victim tree. 6TC, 7TC, 8 TB are seized timber.

Thirty-seven victim trees were reported by Luodong Forest District Office in December 2018. According to census data, the crime scene forest area is 281.03 hectare and the density of *C. taiwanensis* is 16 ± 1.6 individuals/hectare. However, in order to protect suspects’ rights, we took an excess of the maximum possible population size: 10,000 into the calculation. Among 22 samples in this case, 7 succeeded in analysis, which is, by our definition, showing positive result in just 35 PCR cycles. The rest were denoted “Not detected” due to low positive PCR result (all tests comply the standard of accredited laboratory ISO/IEC 17025) or CL < 95%. It is worthwhile to note that seized illegally-felled timber 6TC matched the victim tree 6 TB (CP_I_ = 3.342 × 10^–13^, CPD = 0.999999999999666, CL = 99.9999999%). In addition, seized illegally-felled timber 7TC matched with victim tree 7TA and 7 TB (CP_I_ = 1.631 × 10^–13^, CPD = 0.999999999999837, CL = 99.9999999%), and seized illegally-felled timber 8 TB matched with victim tree 8TA (CP_I_ = 4.468 × 10^–10^, CPD = 0.999999999553151, CL = 99.999532%). In this individual identification system test case (Table 6, Fig. 2, Supplementary Sect. 4), the minimal amount of matching marker was 17 among the positive-matched groups (CL = 99.999532%). The credibility increased along with the matching marker amount. The credibility is dependent on population size and matching marker amount. In addition, while aligning the evidence to the victim individuals, it is a common scenario that the sample DNA might have been degraded. Successful extraction is one of the crucial steps to identify same individual using DNA matching techniques. The extractable DNA in desiccated timber is low in quantity and poor in quality. The extracted DNA can only be used for individual identification using markers developed for specific species. In this regard, SSR marker is a traditional marker for individual identification, which has been widely used in human and gradually extended to other species. All the SSR markers designed in this study are shorter than 300 bp, which would be suitable for amplifying the lysed DNA fragments from desiccated timbers. Although SSR marker has the merits above mentioned, the overall success rate of DNA extraction and genotyping from timber is relatively low (31.81%, 7 out of 22 samples tested successfully). The low quantity and quality of DNA in timber sample might have limited our success rate. An improvement on DNA extraction method would enhance our success rate on timber samples. In addition, increasing the number of SSR markers capable of individual identification would decrease the overall CP_I_ and increase CL. Overall, we provide scientific proof that can be used directly as court evidence in illegal felling cases. This is the first time study reporting the SSR individual identification system which could be applied to various precious species. A warning to forestall illegal felling is the most valuable impact of this study: DNA types of these precious trees have been filed. The illegal felling crime rate is dropping after public propagation of cypress individual identification system. Moreover, the individual identification system would also provide certificate for legal timber trading^21^. This system would also deter dishonest businessman piggybacking illegal material in legal timber auction, which would further forestall illegal logging. In addition, these markers can be also used in population genetic analysis studies that facilitate the conservation and breeding of *C. taiwanensis*.

## Conclusions

In this study, we developed an individual identification system for *C. taiwanensis* and provided the scientific evidence. This methodology can be adopted by the courts to link seized timber and victim trees. The *C. taiwanensis* individual identification system of this study includes 23 gSSR and 12 EST-SSR markers revealing polymorphism. When the 30 non-linkage markers were applied to *C. taiwanensis* identification, the lowest CP_I_ was 5.596 × 10^–12^ and the highest CPD was 0.999999999994404, which was sufficient to identify 18 million random samples of *C. taiwanensis* (CL = 99.99%). While applied in the criminal cases of *C. taiwanensis* illegal logging, this SSR marker system successfully matched five seized illegally-felled timbers to three victim trees with minimal 99.99% CL. To the best of our knowledge, this is the first time the SSR technology is being applied to provide molecular evidence for court conviction on *C. taiwanensis* illegal logging. Our study would provide not only the scientific evidence correlating seized timber and victim tree, but also could inherent unique serial number to identify every single *C. taiwanensis* timber. We demonstrated the feasibility of matching seized/ illegally-felled timber with victim tree by modern SSR technology, which would prevent illegal logging by warning the criminals that the woodland trees could be identified on the basis of molecular level. Additionally, these markers can be also used in population genetic analysis studies that facilitate the conservation and breeding of *C. taiwanensis*.

## Materials and methods

### Developing *C. taiwanensis* individual identification system

#### Library preparation and SSR enrichment

In this study, we constructed both DNA and RNA libraries of *C. taiwanensis* (Fig. 1.). Three DNA libraries were created from individuals of TP (Voucher no. *Chung 2448*) and 100R (Voucher no. *Chung 2603, 2621*) (Supplementary Sect. 1). To build the DNA libraries, genomic DNA was extracted from fresh leaves using the cetyltrimethylammonium bromide (CTAB) method^49^. The quality and concentration of DNA were measured by NanoDrop 2000 (Thermo Fisher Scientific, San Diego, California, USA) and Qubit 2.0 Fluorometer (Thermo Fisher Scientific). From the total genomic DNA, microsatellites enriched in SSR markers was followed the magnetic bead enrichment method of Glenn and Schable^50^. Briefly, DNA was digested using AluI/XmnI and HaeIII/XmnI (New England Biolabs, Ipswich, Massachusetts, USA). The double-stranded SuperSNX linkers (SuperSNX24 Forward: 5′-GTTTAAGGCCTAGCTAGCAGAATC-3′; SuperSNX24 + 4p: 5′-pGATTCTGCTAGCTAGGCCTTAAACAAA-3′) were ligated to the digested DNA fragments. The linker-conjugated DNA fragments were hybridized with Biotin-labeled microsatellite probes containing Mix 2: (AG)_12_, (TG)_12_; Mix 3: (AAC)_6_, (AAG)_8_, (AAT)_12_, (ATC)_8,_ (ACT)_12_; Mix4: (AAAC)_6_, (AAAG)_6_, (AATC)_6_, (AATG)_6_, (ACAG)_6_, (ACCT)_6_, (ACTG)_6_, (ACTC)_6_, (AAAT)_8_, (AACT)_8_, (ACAT)_8_, (AAGT)_8_, and (AGAT)_8_. The SSR hybridized fragments were extracted using Streptavidin M-280 Dynabeads (Invitrogen, Carlsbad, Calsbad, California, USA) and recovered by PCR using the SuperSNX24 Forward primers. The concentration and quality of SSR-enriched libraries were measured by Nanodrop 2000 (Thermo Fisher Scientific, Carlsbad, San Diego, California, USA) and Qubit 2.0 Fluorometer (Thermo Fisher Scientific, USA).

One individual of *C. taiwanensis* (Voucher no.: *Chung 2627*) from XI was used to prepare RNA library. RNA was extracted from fresh leaves by using the CTAB method^51^. The quality and concentration of RNA were measured by NanoDrop 2000 and Qubit 2.0 Fluorometer. The RNA was reverse transcribed into complementary DNA (cDNA) using Ovation RNA-Seq System V2 (NuGEN, San Carlos, California, USA) and the cDNA was quantitated using Nanodrop 2000 and Agilent 2100 Bioanalyzer (Agilent Technologies, Palo Alto, California, USA) by Tri-I Biotech, Inc. (New Taipei City, Taiwan). The cDNA was fragmented by Covaris S220 focused-ultrasonicator (Covaris, Woburm, Massachusetts, USA) and the cDNA library was prepared according to the manual of Ovation Ultralow DR Multiplex System 1–96 (NuGEN).

#### Sequencing and analysis

Three DNA and one RNA libraries were sequenced using the Illumina MiSeq System (2 × 300 bp paired-end; Illumina, San Diego, California, USA) at Tri-I Biotech (New Taipei City, Taiwan). The raw reads were prescreened to remove adapter sequences and reads with greater than 0.1% error or with an average quality less than QV30. High-quality filtered DNA and cDNA reads were merged by CLC Genomics Workbench version 7.5 (QIAGENE, Aarhus, Denmark).

#### SSR screening and primer design

SSRIT was applied to screen the gSSR and EST-SSR containing sequences from contigs. To design gSSR and EST-SSR primers, sequences with at least five di-, tri-, tetra-, penta-, and hexa-nucleotide repeats were selected using BatchPrimer3^52^, with optimized conditions set length at 18–23 bp, melting temperature 45–62 ℃, and a product size of 80–300 bp.

#### Marker validation

A total of 75 markers including 23 gSSR and 12 EST-SSR markers newly designed in this study, and 40 published SSR^4,53,54^ (Supplementary Sect. 2) were subjected to validation test on 96 samples from four *C. taiwanensis* populations (TP, SY, DS and FR, see Supplementary Sect. 1). In addition, we also tested cross-species transferability of the designed gSSR and EST-SSR markers (Supplementary Sect. 3). The samples used in marker validation and cross-species transferability of DNA were extracted using the VIOGENE plant DNA extraction kit (VIOGENE, New Taipei City, Taiwan). The PCR reaction was conducted with a final volume 20 μL containing 2 ng of genomic DNA, 0.25 μL of 10 μM each primer and 10 μL of Q-Amp 2 × Screening Fire Taq Master Mix (Bio-Genesis Technologies, Taipei, Taiwan). The following PCR conditions were used: an initial denaturation of 95 ℃ for 2 min; 30 cycles of 95 ℃ for 45 s, a primer-specific annealing temperature (Tables 1, 2) for 45 s, and 72 ℃ for 45 s; followed by a 15-min extension at 72 ℃ (Tables 1, 2). The amplified products were evaluated on the ABI 3130XL (Applied Biosystems, Waltham, Massachusetts, USA) with GeneScan 500 ROX Size Standard (Applied Biosystems). Fragment size was determined by using GeneMapper version 3.2 (Applied Biosystems).

#### Marker analysis

GenAlex 6.51b2^55^ was used to calculate number of alleles (A), observed heterozygosity (*Ho*), expected heterozygosity (*He*), Hardy–Weinberg equilibrium (HWE) of the newly developed gSSR and EST-SST markers. PowerMarker V3.25^56^ was used to calculate polymorphism information content or power of information content (PIC)^57^. Power of discrimination (PD)^58^, PD = 1 − Σ*P*_*i*_^2^, where *P*_*i*_ is the frequency of genotype *i* . Power of exclusion or probability of exclusion (PE)^58^, PE = *h*^2^[1 − 2* h*(1 − *h*)^2^], where *h* is the frequency of heterozygotes. Probability of identity (P_I_)^59^, P_I_ = 1 − PD. Combined power of discrimination (CPD)^58^, here we calculated CPD of 30 markers. CPD = 1 − [(1 − PD_1_)(1 − PD_2_)…(1 − PD_30_)].Combined probability of identity (CP_I_)^59^. Microsoft Excel (Microsoft Office 2016) was used to calculate PD, PI, PE, CPD, CPI. GENEPOP 4.2^60^ was used to test for linkage disequilibrium.

### Aligning seized timbers to victim trees

Samples from five seized timbers of Taiwan Yilan District Prosecutors Office, six illegally-felled timbers found at crime scene woodland and seven victim trees (Supplementary Sect. 4) were collected. Duplicates of a victim tree (7TA and 7TB) was sourced out in order to ensure the reproducibility of the identical SSR type in individual tree. Two grams of each sample was powdered in liquid nitrogen and the total genomic DNA was extracted following the protocol of VIOGENE plant DNA extraction kit (VIOGENE, New Taipei City, Taiwan). Nineteen non-linkage markers were selected for DNA typing. The sample succeeded in typing were further combined to the aforementioned database to calculation the CP_I_.

## Supplementary Information

Supplementary Information.

## References

[1] Hwang SY, Lin HW, Kuo YS, Lin TP (2001). RAPD variation in relation to population differentiation of *Chamaecyparis formosensis* and *Chamaecyparis taiwanensis*. Bot. Bull. Acad. Sin..

[2] Wang WP, Hwang CY, Lin TP, Hwang SY (2003). Historical biogeography and phylogenetic relationships of the genus *Chamaecyparis* (Cupressaceae) inferred from chloroplast DNA polymorphism. Plant Syst. Evol..

[3] Chen YJ, Chang ST (2017). Distribution and characteristic comparisons of the endemic cypress in Taiwan. Taiwan J. For. Sci..

[4] Huang CJ (2018). Isolation and characterization of SSR and EST-SSR loci in *Chamaecyparis formosensis* (Cupressaceae). Appl. Plant Sci..

[5] Tereba A, Woodward S, Konecka A, Borys M, Nowakowska JA (2017). Analysis of DNA profiles of ash (*Fraxinus excelsior* L.) to provide evidence of illegal logging. Wood Sci. Technol..

[6] Cabral EC (2012). Wood typification by Venturi easy ambient sonic spray ionization mass spectrometry: The case of the endangered Mahogany tree. J. Mass Spectrom..

[7] Kite GC (2010). Dalnigrin, a neoflavonoid marker for the identification of Brazilian rosewood (*Dalbergia nigra*) in CITES enforcement. Phytochemistry.

[8] Dormontt EE (2015). Forensic timber identification: It's time to integrate disciplines to combat illegal logging. Biol. Cons..

[9] Vlam M (2018). Developing forensic tools for an African timber: Regional origin is revealed by genetic characteristics, but not by isotopic signature. Biol. Cons..

[10] Celani CP, Lancaster CA, Jordan JA, Espinoza EO, Booksh KS (2019). Assessing utility of handheld laser induced breakdown spectroscopy as a means of *Dalbergia speciation*. Analyst.

[11] Gasson P (2011). How precise can wood identification be? Wood anatomy’s role in support of the legal timber trade, especially CITES. IAWA J..

[12] Speer JH (2010). Fundamentals of Tree-Ring Research.

[13] McClure PJ, Chavarria GD, Espinoza E (2015). Metabolic chemotypes of CITES protected Dalbergia timbers from Africa, Madagascar, and Asia. Rapid Commun. Mass Spectrom..

[14] Tsuchikawa S, Schwanninger M (2013). A review of recent near-infrared research for wood and paper (Part 2). Appl. Spectrosc. Rev..

[15] Braun, B. Wildlife detector dogs—A guideline on the training of dogs to detect wildlife in trade. *WWF Germany, Berlin*, 1–16 (2013).

[16] Rummel S, Hoelzl S, Horn P, Rossmann A, Schlicht C (2010). The combination of stable isotope abundance ratios of H, C, N and S with 87Sr/86Sr for geographical origin assignment of orange juices. Food Chem..

[17] Hua Q, Barbetti M, Rakowski AZ (2013). Atmospheric radiocarbon for the period 1950–2010. Radiocarbon.

[18] Hollingsworth PM (2009). A DNA barcode for land plants. Proc. Natl. Acad. Sci..

[19] Lowe AJ, Cross HB (2011). The applicat ion of DNA methods to timber tracking and origin verification. Iawa J..

[20] Jobling MA, Gill P (2004). Encoded evidence: DNA in forensic analysis. Nat. Rev. Genet..

[21] Lowe AJ, Wong KN, Tiong YS, Iyerh S, Chew FT (2010). A DNA Method to verify the integrity of timber supply chains; confirming the legal sourcing of merbau timber from logging concession to sawmill. Silvae Genetica.

[22] Dawnay N (2008). A forensic STR profiling system for the Eurasian badger: A framework for developing profiling systems for wildlife species. Forensic Sci. Int. Genet..

[23] Fregeau CJ, Fourney RM (1993). DNA typing with fluorescently tagged short tandem repeats: A sensitive and accurate approach to human identification. Biotechniques.

[24] Varshney RK, Graner A, Sorrells ME (2005). Genic microsatellite markers in plants: features and applications. Trends Biotechnol..

[25] Cho YG (2000). Diversity of microsatellites derived from genomic libraries and GenBank sequences in rice (*Oryza sativa* L.). Theor. Appl. Genet..

[26] Eujayl I, Sorrells M, Baum M, Wolters P, Powell W (2001). Assessment of genotypic variation among cultivated durum wheat based on EST-SSRS and genomic SSRS. Euphytica.

[27] Wang C (2018). Genome survey sequencing of purple elephant grass (*Pennisetum purpureum* Schum ‘Zise’) and identification of its SSR markers. Mol. Breed..

[28] Zhou S (2018). The first Illumina-based de novo transcriptome analysis and molecular marker development in Napier grass (*Pennisetum purpureum*). Mol. Breed..

[29] Liao PC, Lin TP, Hwang SY (2010). Reexamination of the pattern of geographical disjunction of *Chamaecyparis* (Cupressaceae) in North America and East Asia. Bot. Stud..

[30] Schroeder H (2016). Development of molecular markers for determining continental origin of wood from White Oaks (*Quercus* L. sect. *Quercus*). PLoS ONE.

[31] Temnykh S (2001). Computational and experimental analysis of microsatellites in rice (*Oryza sativa* L.): Frequency, length variation, transposon associations, and genetic marker potential. Genome Res..

[32] Liu G, Xie Y, Zhang D, Chen H (2018). Analysis of SSR loci and development of SSR primers in *Eucalyptus*. J. For. Res..

[33] Zhang M, Mao W, Zhang G, Wu F (2014). Development and characterization of polymorphic EST-SSR and genomic SSR markers for Tibetan annual wild barley. PLoS ONE.

[34] Gao L, Tang J, Li H, Jia J (2003). Analysis of microsatellites in major crops assessed by computational and experimental approaches. Mol. Breed..

[35] Kantety RV, La Rota M, Matthews DE, Sorrells ME (2002). Data mining for simple sequence repeats in expressed sequence tags from barley, maize, rice, sorghum and wheat. Plant Mol. Biol..

[36] Sharma R (2016). Genetic diversity estimates point to immediate efforts for conserving the endangered Tibetan sheep of India. Meta Gene.

[37] Ouyang P (2018). Development and characterization of high-throughput EST-Based SSR markers for *Pogostemon cablin* using transcriptome sequencing. Molecules.

[38] Hizume M, Kondo T, Shibata F, Ishizuka R (2001). Flow cytometric determination of genome size in the Taxodiaceae, Cupressaceae sensu stricto and Sciadopityaceae. Cytologia.

[39] Ohri D, Khoshoo TN (1986). Genome size in gymnosperms. Plant Syst. Evol..

[40] Wall, W. *Genetics & DNA Technology: Legal Aspects*. (Routledge-Cavendish, 2002).

[41] Qiu, L. W., Huang, Q. X., Wu, C. C. & Hsieh, H. T. *The Summary of the Fourth Forest Resources Inventory in Taiwan.* (Taipei, 2015).

[42] Degen, B. & Fladung, M. Use of DNA-markers for tracing illegal logging. In *Proceedings of the international workshop “Fingerprinting methods for the identification of timber origins” October*. 8–9 (2007).

[43] Asif M, Cannon CH (2005). DNA extraction from processed wood: A case study for the identification of an endangered timber species (*Gonystylus bancanus*). Plant Mol. Biol. Rep..

[44] Fatima T, Srivastava A, Hanur VS, Rao MS (2018). An effective wood DNA extraction protocol for three economic important timber species of India. Am. J. Plant Sci..

[45] Tnah LH, Lee SL, Ng KKS, Bhassu S, Othman RY (2012). DNA extraction from dry wood of *Neobalanocarpus heimii* (Dipterocarpaceae) for forensic DNA profiling and timber tracking. Wood Sci. Technol..

[46] Dormontt E (2020). Forensic validation of a SNP and INDEL panel for individualisation of timber from bigleaf maple (*Acer macrophyllum* Pursch). Forensic Sci. Int. Genet..

[47] Blais J (2015). Risk of misdiagnosis due to allele dropout and false-positive PCR artifacts in molecular diagnostics: Analysis of 30,769 genotypes. J. Mol. Diagn..

[48] Cummings SM, McMullan M, Joyce DA, van Oosterhout C (2010). Solutions for PCR, cloning and sequencing errors in population genetic analysis. Conserv. Genet..

[49] Doyle, J. J. & Doyle, J. L. A rapid DNA isolation procedure for small quantities of fresh leaf tissue. (1987).

[50] Glenn TC, Schable NA (2005). Isolating microsatellite DNA loci. Methods Enzymol..

[51] Chang S, Puryear J, Cairney J (1993). A simple and efficient method for isolating RNA from pine trees. Plant Mol. Biol. Rep..

[52] You FM (2008). BatchPrimer3: A high throughput web application for PCR and sequencing primer design. BMC Bioinform..

[53] Nakao Y, Iwata H, Matsumoto A, Tsumura Y, Tomaru N (2001). Highly polymorphic microsatellite markers in *Chamaecyparis obtusa*. Can. J. For. Res..

[54] Matsumoto A (2006). Development and polymorphisms of microsatellite markers for hinoki (*Chamaecyparis obtusa*). Mol. Ecol. Notes.

[55] Peakall R, Smouse PE (2012). GenAlEx 6.5: Genetic analysis in Excel. Population genetic software for teaching and research—An update. Bioinformatics.

[56] Liu K, Muse SV (2005). PowerMarker: An integrated analysis environment for genetic marker analysis. Bioinformatics.

[57] Botstein D, White RL, Skolnick M, Davis RW (1980). Construction of a genetic linkage map in man using restriction fragment length polymorphisms. Am. J. Hum. Genet..

[58] Fisher R (1951). Standard calculations for evaluating a blood-group system. Heredity.

[59] Jones DA (1972). Blood samples: Probability of discrimination. J. Forensic Sci. Soc..

[60] Raymond M, Rousset F (1995). GENEPOP (version 1.2): Population genetics software for exact tests and ecumenicism. J. Hered..

