# Supplementary material for: Development and technical application of SSR-based individual identification system for Chamaecyparis taiwanensis against illegal logging convictions
Source: Sci Rep. 2020 Dec 16;10:22095. doi: 10.1038/s41598-020-79061-z (PMC7744516; doi:10.1038/s41598-020-79061-z)
Supplement: Supplementary file 1 — Supplementary Information. [file 41598_2020_79061_MOESM1_ESM.docx]

(Supplementary) Development and technical application of SSR-based individual identification system for *Chamaecyparis taiwanensis*against illegal logging convictions

Chiun-Jr Huang^1,2,3*^, Fang-Hua Chu^1^, Yi-Shiang Huang^4^, Yu-Mei Hung^3^, Yu-Hsin Tseng^2^, Chang-En Pu^5^, Chi-Hsiang Chao^3^, Yu-Shyang Chou^3^, Shau-Chian Liu^6^, Ya Ting You^2^, Shuo-Yu Hsu^1^, Hsiang-Chih Hsieh^1^, Cheng Te Hsu^7^, Meng-Yi Chen^3^, Ting-An Lin^3^, Hsin-Yi Shyu^3^, Yu-Ching Tu^3^ and Chi-Tsong Chen^3*^

^1^School of Forestry and Resource Conservation, National Taiwan University, Taipei 10617, Taiwan. ^2^Biodiversity Research Center, Academia Sinica, Taipei 11529, Taiwan. ^3^Department of Forensic Science, Investigation Bureau, Ministry of Justice, New Taipei City 23149, Taiwan. ^4^Institute of Biological Chemistry, Academia Sinica, Taipei, 11529, Taiwan. ^5^Department of Research Committee, Investigation Bureau, Ministry of Justice, New Taipei City 23149, Taiwan. ^6^Department of Applied Science, National Taitung University, Taitung 95092, Taiwan. ^7^Hualien Forest District Office, Forestry Bureau, Council of Agriculture, Hualien 97051, Taiwan. Correspondence and requests for materials should be addressed to C.J.H. (email: [d04625001@ntu.edu.tw](mailto:d04625001@ntu.edu.tw)) or C.T.C. (email: [chen33039@gmail.com](mailto:chen33039@gmail.com))

**Supplementary section 1: Voucher information**

The voucher information of individuals sampled in this study is summarized in Table S1. All voucher specimens are deposited in the Herbarium of the Biodiversity Research Center (HAST), Academia Sinica, Taipei, Taiwan.

**Table S1:** Voucher information for *C. taiwanensis* and *C. formosensis* individuals.

| Species | Application | Voucher no. | Collection locality | Geographic coordinates | Population code | *N* |
| --- | --- | --- | --- | --- | --- | --- |
| *C. taiwanensis* | Genomic library for NGS | *Chung 2448* | Taipingshan Forest Recreation Area, Datong Township, Yilan County 267, Taiwan. | 24°29'42.27’’N, 121°32'06.25’’E. | TP | 1 |
|  | Genomic library for NGS | *Chung 2603, 2621* | No. 100 Forest Rd., Jianshi Township, Hsinchu County 313, Taiwan. | 24°35'30.23’’N, 121°25'12.32’’E. | 100R | 2 |
|  | cDNA library for NGS | *Chung 2627* | XITOU Nature Education Area, Lugu Township, Nantou 558, Taiwan. | 23°39'38.12’’N, 120°47'54.41’’E. | XI | 1 |
|  | Test genetic diversity | *Chung 2427, 2428, 2429, 2431, 2435, 2436, 2437, 2438, 2439, 2440, 2441, 2442, 2443, 2444, 2445, 2446, 2447, 2448, 2449, 2450, 2451, 2452, 2453, 2454, 2455* | Taipingshan Forest Recreation Area, Datong Township, Yilan County 267, Taiwan. | 24°29'42.27’’N, 121°32'06.25’’E. | TP | 25 |
|  |  | *Chung 2701, 2702, 2703, 2704, 2705, 2706, 2707, 2708, 2709, 2710, 2711, 2712, 2714, 2715, 2716, 2717, 2718, 2720, 2721, 2722, 2723, 2724, 2725, 2726, 2727, 2728, 2729, 2730, 2731* | Siyuan Wind Gap, Datong Township, Yilan County 267, Taiwan. | 24°23'49.80’’N, 121°21'11.55’’E. | SY | 29 |
|  |  | *Chung 3222, 3227, 3228, 3229, 3233, 3240, 3241, 3242, 3245, 3247, 3248, 3250, 3255, 3301, 3315, 3317, 3318, 3324, 3325, 4001, 4004* | Dasyueshan Forest Recreation Area | 24°13'9.59’’N, 120°53'9.06’’E. | DS | 21 |
|  |  | *Chung 4430, 4432, 4433, 4435, 4530, 4537, 4541, 4542, 4543, 4544, 4545, 4546, 4547, 4549, 4550, 4553, 4554, 4557, 4558, 4563, 4568* | No.160 Forest Road, Fuxing Dist, Taoyuan City 336, Taiwan. | 24°32'20.62"N, 121°22'37.56"E. | FR | 21 |
| *C. formosensis* | Test cross-amplification | *Chung 4439* | Qilan Forest Recration Area, Fuxing Dist, Taoyuan City 336, Taiwan. | 24°35'14.89’’N, 121°26'07.80’’E. | QL | 1 |
|  |  | *Chung 4901* | No. 20 provincial hwy., Haiduan Township, Taitung Country 957, Taiwan. | 120°59'34.00’’N, 23°14'46.51’’E. | PH | 1 |
|  |  | *Chung 4907* | Yanping Forest Rd., Yanping Township, Taitung Country 953, Taiwan. | 120°57'33.84"N, 22°56'0.24"E. | YP | 1 |
|  |  | *Chung 5102* | Alishan Forest Recration Area, Alishan Township, Chiayi Country 605, Taiwan. | 120°48'52.97"N, 23°30'37.24"E. | AL | 1 |
|  |  | *Chung 5308* | Malibasi Forest Rd., Fenglin Township, Hualien County 975, Taiwan. | 121°18'47.04"N, 23°44'18.30"E. | ML | 1 |
|  |  | *Chung 5312* | Guangfu Forest Rd., Guangfu Township, Hualien County 976, Taiwan. | 121°18'34.98’’N, 23°37'26.22’’E. | GF | 1 |
|  |  | *Chung 5351* | Papaya mountain, Xiulin Township, Hualien County 972, Taiwan. | 121°23'31.70’’N, 24°02'32.33’’E. | PP | 1 |
|  |  | *Chung 5378* | Changlin Forest Rd., Zhuoxi Township, Hualien County 982, Taiwan. | 121°12'18.89’’N, 23°14'46.55’’E. | CG | 1 |

*N* = number of individuals

**Supplementary section 2: Published markers test in *C. taiwanensis***

Forty-one published SSR markers for *C. obtusa* and *C. formosensis*^1-3^ were tested against *C. taiwanensis* (for details of the selected 41 markers see Table S2). All markers had poor results such as null PCR product, multiple PCR product, or single genotype in the pretest. The PCR success rate is considerably lower than published studies (62.96%^3^ and 50.00%^4^) because of pursuing clean and polymorphism in PCR products. Moreover, the result also dictates the existence of molecule discrepancy among different cypresses, which hurdles the direct application of markers from other species to *C. taiwanensis*.

**Table S2:** Published markers applied to this study design. These markers are from *Chamaecyparis obtusa*^1,2^, and *C.* *formosensis*^3^.

| Locus | Forward sequence | Reverse sequence | *Ta* (℃) |
| --- | --- | --- | --- |
| Co31 | AACAAATAGGCACCCAACTTC | GATGGTGAGATGAGGGAGG | 45 |
| Co66 | CTAGGAGCCAATCTAAGACTTCTC | TGACAATGAAATCCTACAAGACC | 45 |
| Co67 | CTCAAATAACTACCCAAACACTC | TCCAATGCCTTACAAAGC | 50 |
| Co69 | TATATTGGCTCAAGGTGGGT | AATCTGAGAGCTGCAAGGAA | 50 |
| Co88 | GAAGGTAGTTGGTAGAAGTATTAG | ACACATGGAAGCAATTATAC | 56 |
| Co93 | CAAGCAGCTACAACAAAGAATGAC | AGCAAGAAGGTGAAAGTTATGAGG | 56 |
| Co115 | AATACACAATCGAATCAATC | TGCAGGACTGGTTTTAG | 56 |
| Co118 | CTTGATTTATGATAGATTTGTTG | GGCATTAGACTTAGTGGATT | 56 |
| Co144 | CTTGTACTTGTTGGTTGTG | ATTTAGGTCTCTTTATAGTCCTT | 56 |
| Cos0319 | GCTACTAATAGAGGGAGGGA | AATAAAATGACAAGGGGATA | 55 |
| Cos1526 | AGGAGCGGGTAGACATTATTCAGTC | CCTATAGTATGCCCAAATCTTGTTGC | 55 |
| Cos1536 | GATCAAGTTCATAAGTTAGGATTG | GTTTCTTTATGGAGGAGAAAGTGACAA | 55 |
| Cos1761 | CTTCCTGGTATGAATCAACT | ACGGACAAACATCTTCTCT | 55 |
| Cos1874 | TAGTGCTAGGGATTGATGAG | GACCACCTTGAACTCTAACC | 49 |
| Cos1951 | AAGTGAAATAAGTCCTCATCAAATCC | GTTTCTTCATGAACATAACCAACACTGCC | 55 |
| Cos1991 | AATTGCGTGATATGAGTGTGTGGC | CAGAGGGATAATTCACTTGTTGAGGG | 63 |
| Cos2126 | GCGAAATCAAGTGGAGTC | AAGAGGGAGGATGTTGTAATAC | 45 |
| Cos2165 | GGTATCAACAAATCATCAAATGC | GTTTCTTCAAGTTTGGAATCAAGGGAG | 55 |
| Cos2224 | TGGTGATGTCAGGGTTAGAG | GGCTAGGATGGGAGGG | 63 |
| Cos2590 | CAACCCACATCACCACCTGAC | TGGATGAGGAGAGGCAGTTGTAGTTA | 63 |
| Cos2610 | GATCTATGTCTCTTCGGTACTATT | TTACACAATGGGATGATGA | 55 |
| Cos2619 | CCCTACCATTACCTGTTATATATGTGTG | GCTATGGTTAGGATTTGGGACTG | 56 |
| Cos2667 | TTCCAATTATAACTCATTATGGG | TCCTTTATTCCTTTAATCTTGTTT | 53 |
| Cos2680 | CTTGCATGTGTTTTTACAAGCTAAG | TTAGTATCATGTTCCAGGTTATTCTCTC | 50 |
| Cred35 | GGAGAAAGGAGTGTCACAAG | AACTCATTCCTTCTCCCTCT | TD58-55 |
| Cred211 | AAAAATATCAAGCAGATTACCTCTA | TCTTTCTTATCTTTTTCTTT | 49 |
| Cred220 | CACTGATCTTATGGAGACCATACT | ATCCATCCCTACAATCCTTAC | 49 |
| Cred225 | GGGTTTCTCTCCTACCATTT | TGAGATGCATTAGATTAGAGG | 57 |
| Cred226 | CTAGCTCTTCTTCTGGTTGC | AAAGATATTGATAAAGCAGAAACA | 57 |
| Cred229 | GGAGAAAGGAGTGTCACAAG | CTCTATTTATATCCCATTTCCTCT | 49 |
| Cred231 | TACTCAGAAGTGACAACACAAA | GGCATGTATGAATCTTGTGA | 57 |
| Cred236 | GGGCATACACTCCACTTAAA | GGATTTGTGTTTCCATAAGGT | 57 |
| Cred248 | TCCATTTCCTAGACTTACCG | GCACTACCCATCAGTTATCAA | 57 |
| Cred249 | AGCACACTTAATAATAGGATAGA | TGATTTCAATGAGGTATTTCC | 57 |
| Cred253 | TTTCCTCAGATCTTGCTTA | AAGGAAAGAGGAAACCTGAA | 49 |
| Cred260 | CCTTTCAAACATACACTCAAA | GCCCAACATGTATGAAGTTT | 45 |
| Cred264 | TTCTATAATAGGGGCAGCTT | CCTTCTAAAAGTAGAACCCAAG | 56 |
| Cred276 | CCTTCCTAAGCGGTTCGTG | CCATCCATCCCTTTCTTTCA | 56 |
| Cred277 | CCCTTCTTCGCCATCTTCTT | CAGAAAGACAAACCGAAAGACA | 62 |
| Cred280 | GCAATGTCTTGAAGCGCTTATC | ATACCAATTCAACAATTCATCACAA | 56 |
| Cred299 | GCCATAGCTACCACCACCAC | AGATGTCACTGGCCCTAATGG | 62 |

**Supplementary section 3. Cross-species transferability**

The cross-species transferability of the 23 gSSR and 12 EST-SSR markers was evaluated with *Chamaecyparis formosensis* (Table S3)*.* Locality and voucher information for the population are presented in Supplementary section 1. Of these markers, 15 in gSSR (65.2%) and nine in EST-SSR (75.0%) were successfully amplified in all eight individuals from eight populations. In addition, EST-SSR markers showed higher transferability than gSSR markers as expected, which is the manifestation of nature conservation on transcribed regions.

**Table S3:** Cross-amplification results for 23 gSSR and 12 EST-SSR loci developed in *C. taiwanensis* in eight populations of *C. fo**rmosensis*.

| Locus | Type | QL | PH | YP | AL | ML | GF | PP | CG | **+ / -** |
| --- | --- | --- | --- | --- | --- | --- | --- | --- | --- | --- |
| CoTW76 | gSSR | + | + | + | + | + | + | + | + | **8 / 0** |
| CoTW77 | gSSR | + | + | + | + | + | + | + | + | **8 / 0** |
| CoTW99 | gSSR | + | + | + | + | + | + | + | + | **8 / 0** |
| CoTW314 | gSSR | + | + | + | + | + | + | + | + | **8 / 0** |
| CoTW330 | gSSR | + | + | + | + | + | + | + | + | **8 / 0** |
| CoTW337 | gSSR | + | - | - | - | - | - | + | + | **3 / 5** |
| CoTW349 | gSSR | + | + | + | + | + | + | + | + | **8 / 0** |
| CoTW495 | gSSR | + | + | + | + | + | + | + | + | **8 / 0** |
| CoTW531 | gSSR | + | + | + | + | + | + | + | + | **8 / 0** |
| CoTW539 | gSSR | + | + | + | + | + | + | + | + | **8 / 0** |
| CoTW545 | gSSR | + | + | + | + | + | + | + | + | **8 / 0** |
| CoTW554 | gSSR | + | + | + | + | + | + | + | + | **8 / 0** |
| CoTW556 | gSSR | + | + | + | + | + | + | + | + | **8 / 0** |
| CoTW559 | gSSR | + | + | + | + | + | + | + | + | **8 / 0** |
| CoTW561 | gSSR | + | + | + | + | - | - | + | + | **6 / 2** |
| CoTW582 | gSSR | + | + | + | + | - | + | + | + | **7 / 1** |
| CoTW585 | gSSR | + | + | + | + | - | + | + | + | **7 / 1** |
| CoTW588 | gSSR | + | + | + | + | + | - | + | + | **7 / 1** |
| CoTW595 | gSSR | - | - | - | + | - | - | + | + | **3 / 5** |
| CoTW597 | gSSR | - | - | - | - | - | - | - | - | **0 / 8** |
| CoTW598 | gSSR | - | - | - | - | - | - | - | - | **0 / 8** |
| CoTW599 | gSSR | + | + | + | + | + | + | + | + | **8 / 0** |
| CoTW600 | gSSR | + | + | + | + | + | + | + | + | **8 / 0** |
| CoTW383 | EST-SSR | + | + | + | + | + | + | + | + | **8 / 0** |
| CoTW407 | EST-SSR | + | + | + | + | + | + | + | + | **8 / 0** |
| CoTW409 | EST-SSR | + | + | + | + | + | + | + | + | **8 / 0** |
| CoTW420 | EST-SSR | + | + | + | + | + | + | + | + | **8 / 0** |
| CoTW424 | EST-SSR | - | - | + | - | - | - | - | - | **1 / 7** |
| CoTW502 | EST-SSR | + | + | + | + | + | + | + | + | **8 / 0** |
| CoTW504 | EST-SSR | + | + | + | - | - | - | + | + | **5 / 3** |
| CoTW511 | EST-SSR | + | + | + | + | + | + | + | + | **8 / 0** |
| CoTW513 | EST-SSR | + | + | + | + | + | + | + | + | **8 / 0** |
| CoTW514 | EST-SSR | - | + | + | + | - | - | + | - | **4 / 4** |
| CoTW528 | EST-SSR | + | + | + | + | + | + | + | + | **8 / 0** |
| CoTW581 | EST-SSR | + | + | + | + | + | + | + | + | **8 / 0** |

+: successful amplification; -: failed amplification.

**Supplementary section 4: Criminal case test**

The voucher information for seized timbers and victim tree in this case is shown in Table S4. The sample photos are shown in Figure 2. Timbers were seized by Yilan District Prosecutors Office and dispatched to Forensic DNA Lab. (ISO/IEC 17025 accredited) of Department of Forensic Science, Ministry of Justice Investigation Bureau for matching. The timbers and DNA samples are designated for permanent retention in MJIB forensic science labs (MJIB-DNA-1080413 combine 1080328).

**Table S4:** Voucher information for seized timbers and victim tree in this case.

| Sample type | Sample name in lab | Collection locality | Match Group | Voucher |
| --- | --- | --- | --- | --- |
| Seized timber | 1T | Evidence room |  | 1080328-1T |
| Seized timber | 2T | Evidence room |  | 1080328-2T |
| Seized timber | 3T | Evidence room |  | 1080328-3T |
| Seized timber | 4T | Evidence room |  | 1080328-4T |
| Seized timber | 5T | Evidence room |  | 1080328-5T |
| Illegally-felled timber | 6TC | 24°37'36.96’’N, 121°27'27.72’’E. | 6TB, 6TC | 1080328-6TC |
| Illegally-felled timber | 7TC | 24°37'36.96’’N, 121°27'27.72’’E. | 7TA, 7TB, 7TC | 1080328-7TC |
| Illegally-felled timber | 8TB | 24°37'38.35’’N, 121°27'27.79’’E. | 8TA, 8TB | 1080328-8TB |
| Illegally-felled timber | 9TC | 24°38'07.45’’N, 121°27'25.48’’E. |  | 1080328-9TC |
| Illegally-felled timber | 10TC | 24°38'09.61’’N, 121°27'30.04’’E. |  | 1080328-10TC |
| Illegally-felled timber | 11TC | 24°37'35.33’’N, 121°27'27.50’’E. |  | 1080328-11TC |
| Victim tree | 6TA | 24°37'36.96’’N, 121°27'27.72’’E. |  | 1080328-6TA |
| Victim tree | 6TB | 24°37'36.96’’N, 121°27'27.72’’E. | 6TB, 6TC | 1080328-6TB |
| Victim tree | 7TA, 7TB | 24°37'36.96’’N, 121°27'27.72’’E. | 7TA, 7TB, 7TC | 1080328-7TA  1080328-7TB |
| Victim tree | 8TA | 24°37'38.35’’N, 121°27'27.79’’E. | 8TA, 8TB | 1080328-8TA |
| Victim tree | 9TA | 24°38'07.45’’N, 121°27'25.48’’E. |  | 1080328-9TA |
| Victim tree | 9TB | 24°38'07.45’’N, 121°27'25.48’’E. |  | 1080328-9TB |
| Victim tree | 10TA | 24°38'09.61’’N, 121°27'30.04’’E. |  | 1080328-10TA |
| Victim tree | 10TB | 24°38'09.61’’N, 121°27'30.04’’E. |  | 1080328-10TB |
| Victim tree | 11TA | 24°37'35.33’’N, 121°27'27.50’’E. |  | 1080328-11TA |
| Victim tree | 11TB | 24°37'35.33’’N, 121°27'27.50’’E. |  | 1080328-11TB |

**References**

1 Nakao, Y., Iwata, H., Matsumoto, A., Tsumura, Y. & Tomaru, N. Highly polymorphic microsatellite markers in *Chamaecyparis obtusa*. *Canadian journal of forest research* **31**, 2248-2251, doi:10.1139/cjfr-31-12-2248 (2001).

2 Matsumoto, A. *et al.* Development and polymorphisms of microsatellite markers for hinoki (*Chamaecyparis obtusa*). *Molecular Ecology Notes* **6**, 310-312, doi:10.1111/j.1471-8286.2006.01212.x (2006).

3 Huang, C. J. *et al.* Isolation and characterization of SSR and EST-SSR loci in *Chamaecyparis formosensis* (Cupressaceae). *Applications in plant sciences* **6**, e01175, doi:10.1002/aps3.1175 (2018).

4 Kim, Y. M., Shin, Y. S. & Jeong, J. H. Development and characterization of microsatellite primers for *Chamaecyparis obtusa* (Cupressaceae). *Applications in plant sciences* **4**, 1500136 (2016).
